# 2,2,2-Trifluoro-*N*-(4-methyl-2-oxo-2*H*-chromen-7-yl)acetamide

**DOI:** 10.1107/S1600536812009634

**Published:** 2012-03-10

**Authors:** Hong-Da Li, Bing-Zhu Yin

**Affiliations:** a Key Laboratory of Natural Resources of Changbai Mountain & Functional Molecules (Yanbian University), Ministry of Eduction, Yanji 133002, People’s Republic of China

## Abstract

In the title mol­ecule, C_12_H_8_F_3_NO_3_, the trifluoro­methyl group is rotationally disordered over three orientations in a 0.5:0.3:0.2 ratio. In the crystal, N—H⋯O hydrogen bonds link the mol­ecules related by translation into chains along the *c* axis. The crystal packing exhibits π–π inter­actions between the pyran rings of neighboring mol­ecules [centroid–centroid distance = 3.462 (4) Å] and short C⋯O contacts of 3.149 (4) Å.

## Related literature

For applications of coumarin derivatives, see: Li *et al.* (2012 ▶). For potential applications of the title compound as a fluorescent probe for cyanide, see: Li *et al.* (2011 ▶).
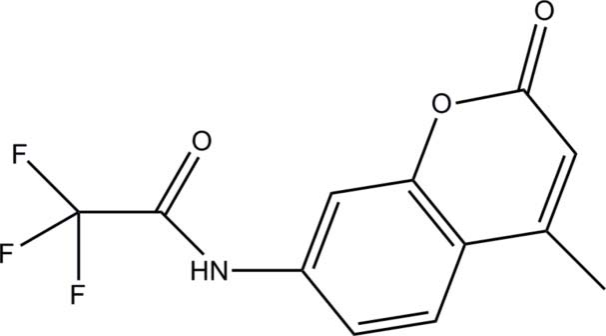


## Experimental

### 

#### Crystal data

C_12_H_8_F_3_NO_3_
*M* *_r_* = 271.19
Triclinic, 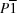

*a* = 8.4897 (17) Å
*b* = 8.5777 (17) Å
*c* = 9.3677 (19) Å
α = 89.36 (3)°
β = 68.27 (3)°
γ = 65.12 (3)°
*V* = 566.3 (2) Å^3^
*Z* = 2
Mo *K*α radiation
μ = 0.15 mm^−1^
*T* = 293 K
0.33 × 0.25 × 0.22 mm

#### Data collection

Rigaku R-AXIS RAPID diffractometer
Absorption correction: multi-scan (*ABSCOR*; Higashi, 1995 ▶) *T*_min_ = 0.954, *T*_max_ = 0.968
5577 measured reflections
2558 independent reflections
1660 reflections with *I* > 2σ(*I*)
*R*_int_ = 0.025

#### Refinement

*R*[*F* ^2^ > 2σ(*F* ^2^)] = 0.046
*wR*(*F* ^2^) = 0.143
*S* = 1.01
2558 reflections
227 parameters
84 restraints
H-atom parameters constrained
Δρ_max_ = 0.32 e Å^−3^
Δρ_min_ = −0.29 e Å^−3^

### 

Data collection: *RAPID-AUTO* (Rigaku, 1998 ▶); cell refinement: *RAPID-AUTO*; data reduction: *CrystalStructure* (Rigaku/MSC and Rigaku, 2002 ▶); program(s) used to solve structure: *SHELXS97* (Sheldrick, 2008 ▶); program(s) used to refine structure: *SHELXL97* (Sheldrick, 2008 ▶); molecular graphics: *SHELXTL* (Sheldrick, 2008 ▶); software used to prepare material for publication: *SHELXL97*.

## Supplementary Material

Crystal structure: contains datablock(s) global, I. DOI: 10.1107/S1600536812009634/cv5256sup1.cif

Structure factors: contains datablock(s) I. DOI: 10.1107/S1600536812009634/cv5256Isup2.hkl

Supplementary material file. DOI: 10.1107/S1600536812009634/cv5256Isup3.cml

Additional supplementary materials:  crystallographic information; 3D view; checkCIF report

## Figures and Tables

**Table 1:** Hydrogen-bond geometry (Å, °)

*D*—H⋯*A*	*D*—H	H⋯*A*	*D*⋯*A*	*D*—H⋯*A*
N1—H1⋯O2^i^	0.86	2.05	2.8960 (19)	170

Symmetry code: (i) 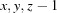.

**Fig. 1.**
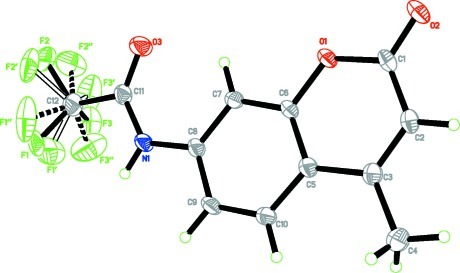
The molecular structure of (I) with the atomic numbering. Displacement ellipsoids are drawn at the 30% probalility level.

## supplementary crystallographic information

### Comment

Coumarins, with the structure of benzopyrone, have many advantages including high fluorescence quantum yield, large Stokes shift, excellent light stability, and low toxicity. Therefore, coumarin derivatives have been used as fluorescent probes of pH, for detection of nitric oxide, nitroxide, and hydrogen peroxide so far. Moreover, coumarin derivatives have served as good chemosensors of anions including cyanide, fluoride, pyrophosphate, acetate, benzoate, and dihydrogenphosphate as well as various metal ions comprised of Hg (II), Cu(II), Zn(II), Ni(II), Ca(II), Pb(II), Mg(II), Fe(III), Al (III), Cr(III), and Ag(I) (Li *et al.*, 2012). Herein, we report the crystal structure of the title compound (I), a potential fluorescent probe for cyanide (Li *et al.*, 2011).

In (I) (Fig. 1), all bond lengths and angles are normal. Intermolecular N—H···O hydrogen bonds (Table 1) link the molecules related by translation along axis *c* into chains. The crystal packing exhibits π···π interactions between the pyran rings from the neighboring molecules [centroid-centroid distance of 3.462 (4) Å] and short C···O contacts of 3.149 (4) Å.

### Experimental

A solution of 7-amino-4-methylcoumarin (100 mg, 0.57 mmol) and trifluoroacetic anhydride (360 mg, 1.70 mmol) was stirred in THF (5 ml) at room temperature for 1 h under N_2_. Then the mixture was concentrated and the solid was recrystallized from THF to give the title compound (113.4 mg), yield 73.3%. Crystals suitable for single-crystal X-ray diffraction were grown by slow evaporation of a mixture of tetrahydrofuran and petroleum (60–90 °C) at room temperature.

### Refinement

C-bound H-atoms were placed in calculated positions (C—H 0.93 and 0.96 Å) and were included in the refinement in the riding model with *U*_iso_(H) = 1.5 or 1.2 *U*_eq_(C). N-bound atom H1 was placed in calculated position with N-H = 0.86 Å and refined with *U*_iso_(H) = 1.2 *U*_eq_(N). Trifluoromethyl group was treated as rotationally disordered over three orientations with the occupancies refined in the initial cycles, but in the final cycle they were fixed to 0.5, 0.3 and 0.2, respectively.

### Crystal data

**Table:**

C_12_H_8_F_3_NO_3_	*Z* = 2
*M**_r_* = 271.19	*F*(000) = 276
Triclinic, *P*1	*D*_x_ = 1.590 Mg m^−^^3^
Hall symbol: -P 1	Mo *K*α radiation, λ = 0.71073 Å
*a* = 8.4897 (17) Å	Cell parameters from 4341 reflections
*b* = 8.5777 (17) Å	θ = 3.3–27.7°
*c* = 9.3677 (19) Å	µ = 0.15 mm^−^^1^
α = 89.36 (3)°	*T* = 293 K
β = 68.27 (3)°	Block, colourless
γ = 65.12 (3)°	0.33 × 0.25 × 0.22 mm
*V* = 566.3 (2) Å^3^	

### Data collection

**Table:**

Rigaku R-AXIS RAPID diffractometer	2558 independent reflections
Radiation source: fine-focus sealed tube	1660 reflections with *I* > 2σ(*I*)
Graphite monochromator	*R*_int_ = 0.025
ω scans	θ_max_ = 27.5°, θ_min_ = 3.3°
Absorption correction: multi-scan (*ABSCOR*; Higashi, 1995)	*h* = −10→11
*T*_min_ = 0.954, *T*_max_ = 0.968	*k* = −9→11
5577 measured reflections	*l* = −12→12

### Refinement

**Table:**

Refinement on *F*^2^	Primary atom site location: structure-invariant direct methods
Least-squares matrix: full	Secondary atom site location: difference Fourier map
*R*[*F*^2^ > 2σ(*F*^2^)] = 0.046	Hydrogen site location: inferred from neighbouring sites
*wR*(*F*^2^) = 0.143	H-atom parameters constrained
*S* = 1.01	*w* = 1/[σ^2^(*F*_o_^2^) + (0.0898*P*)^2^] where *P* = (*F*_o_^2^ + 2*F*_c_^2^)/3
2558 reflections	(Δ/σ)_max_ = 0.043
227 parameters	Δρ_max_ = 0.32 e Å^−^^3^
84 restraints	Δρ_min_ = −0.29 e Å^−^^3^

### Special details

**Table:**

Experimental. (See detailed section in the paper)
Geometry. All e.s.d.'s (except the e.s.d. in the dihedral angle between two l.s. planes) are estimated using the full covariance matrix. The cell e.s.d.'s are taken into account individually in the estimation of e.s.d.'s in distances, angles and torsion angles; correlations between e.s.d.'s in cell parameters are only used when they are defined by crystal symmetry. An approximate (isotropic) treatment of cell e.s.d.'s is used for estimating e.s.d.'s involving l.s. planes.
Refinement. Refinement of *F*^2^ against ALL reflections. The weighted *R*-factor *wR* and goodness of fit *S* are based on *F*^2^, conventional *R*-factors *R* are based on *F*, with *F* set to zero for negative *F*^2^. The threshold expression of *F*^2^ > σ(*F*^2^) is used only for calculating *R*-factors(gt) *etc*. and is not relevant to the choice of reflections for refinement. *R*-factors based on *F*^2^ are statistically about twice as large as those based on *F*, and *R*- factors based on ALL data will be even larger.

### Fractional atomic coordinates and isotropic or equivalent isotropic displacement parameters (Å^2^)

**Table:**

	*x*	*y*	*z*	*U*_iso_*/*U*_eq_	Occ. (<1)
C1	0.2553 (3)	0.1720 (2)	0.69632 (18)	0.0411 (4)	
C2	0.2740 (3)	0.0012 (2)	0.66094 (19)	0.0428 (5)	
H2	0.2863	−0.0729	0.7336	0.051*	
C3	0.2746 (3)	−0.0569 (2)	0.52793 (19)	0.0378 (4)	
C4	0.2947 (4)	−0.2365 (3)	0.4954 (2)	0.0558 (6)	
H4A	0.3023	−0.2943	0.5827	0.084*	
H4B	0.4080	−0.3019	0.4038	0.084*	
H4C	0.1866	−0.2284	0.4789	0.084*	
C5	0.2568 (3)	0.0591 (2)	0.41442 (17)	0.0340 (4)	
C6	0.2416 (2)	0.2238 (2)	0.44841 (17)	0.0317 (4)	
C7	0.2285 (3)	0.3436 (2)	0.34783 (17)	0.0345 (4)	
H7	0.2167	0.4531	0.3753	0.041*	
C8	0.2336 (3)	0.2953 (2)	0.20462 (17)	0.0326 (4)	
C9	0.2482 (3)	0.1313 (2)	0.16628 (18)	0.0411 (5)	
H9	0.2504	0.1001	0.0706	0.049*	
C10	0.2595 (3)	0.0160 (2)	0.26924 (19)	0.0406 (4)	
H10	0.2690	−0.0927	0.2423	0.049*	
C11	0.2084 (3)	0.5682 (2)	0.10611 (19)	0.0367 (4)	
C12	0.2258 (3)	0.6488 (3)	−0.0436 (2)	0.0470 (5)	
F1	0.1414 (13)	0.6068 (13)	−0.1225 (11)	0.0525 (18)	0.50
F2	0.1399 (19)	0.8175 (10)	−0.0063 (12)	0.065 (3)	0.50
F3	0.4003 (12)	0.5937 (15)	−0.1389 (9)	0.068 (3)	0.50
F1'	0.215 (4)	0.570 (3)	−0.150 (3)	0.115 (8)	0.30
F2'	0.087 (3)	0.821 (3)	−0.016 (2)	0.078 (6)	0.30
F3'	0.383 (2)	0.669 (3)	−0.096 (3)	0.098 (6)	0.30
F1''	0.102 (3)	0.685 (4)	−0.094 (3)	0.108 (10)	0.20
F2''	0.273 (6)	0.776 (3)	−0.0344 (18)	0.090 (6)	0.20
F3''	0.394 (5)	0.524 (2)	−0.166 (2)	0.104 (9)	0.20
N1	0.2276 (2)	0.40564 (18)	0.09096 (15)	0.0368 (4)	
H1	0.2373	0.3638	0.0034	0.044*	
O1	0.2404 (2)	0.27853 (15)	0.58734 (12)	0.0395 (3)	
O2	0.2518 (3)	0.23117 (19)	0.81527 (14)	0.0599 (5)	
O3	0.1836 (3)	0.65505 (17)	0.22024 (15)	0.0554 (4)	

### Atomic displacement parameters (Å^2^)

**Table:**

	*U*^11^	*U*^22^	*U*^33^	*U*^12^	*U*^13^	*U*^23^
C1	0.0547 (12)	0.0453 (10)	0.0236 (7)	−0.0192 (9)	−0.0200 (8)	0.0085 (6)
C2	0.0605 (13)	0.0417 (10)	0.0304 (8)	−0.0229 (9)	−0.0231 (8)	0.0157 (7)
C3	0.0504 (12)	0.0351 (9)	0.0319 (8)	−0.0214 (8)	−0.0184 (8)	0.0117 (7)
C4	0.0946 (19)	0.0414 (11)	0.0499 (10)	−0.0384 (12)	−0.0391 (12)	0.0206 (8)
C5	0.0453 (11)	0.0336 (8)	0.0280 (7)	−0.0194 (8)	−0.0179 (7)	0.0090 (6)
C6	0.0415 (10)	0.0343 (8)	0.0222 (7)	−0.0165 (7)	−0.0162 (7)	0.0043 (6)
C7	0.0512 (11)	0.0293 (8)	0.0292 (7)	−0.0202 (8)	−0.0200 (7)	0.0070 (6)
C8	0.0444 (10)	0.0330 (8)	0.0267 (7)	−0.0192 (7)	−0.0188 (7)	0.0096 (6)
C9	0.0690 (14)	0.0402 (9)	0.0301 (8)	−0.0298 (9)	−0.0302 (9)	0.0096 (7)
C10	0.0655 (13)	0.0327 (8)	0.0356 (8)	−0.0264 (9)	−0.0278 (9)	0.0088 (7)
C11	0.0477 (11)	0.0370 (9)	0.0359 (8)	−0.0234 (8)	−0.0227 (8)	0.0146 (7)
C12	0.0608 (15)	0.0455 (11)	0.0448 (10)	−0.0288 (10)	−0.0262 (11)	0.0207 (8)
F1	0.078 (5)	0.055 (4)	0.056 (3)	−0.038 (4)	−0.052 (3)	0.037 (3)
F2	0.102 (8)	0.032 (3)	0.066 (3)	−0.026 (4)	−0.043 (5)	0.028 (2)
F3	0.049 (3)	0.093 (8)	0.056 (4)	−0.035 (5)	−0.011 (3)	0.032 (5)
F1'	0.23 (2)	0.080 (10)	0.067 (9)	−0.075 (15)	−0.088 (15)	0.039 (7)
F2'	0.067 (7)	0.073 (8)	0.070 (6)	−0.007 (4)	−0.030 (4)	0.040 (5)
F3'	0.056 (7)	0.112 (15)	0.127 (15)	−0.047 (10)	−0.030 (9)	0.076 (11)
F1''	0.078 (10)	0.14 (2)	0.119 (15)	−0.043 (14)	−0.058 (10)	0.089 (16)
F2''	0.175 (18)	0.073 (11)	0.063 (7)	−0.083 (12)	−0.057 (12)	0.038 (7)
F3''	0.135 (16)	0.072 (9)	0.044 (5)	−0.035 (10)	0.012 (7)	0.007 (6)
N1	0.0603 (11)	0.0358 (7)	0.0268 (6)	−0.0268 (7)	−0.0245 (7)	0.0120 (5)
O1	0.0628 (9)	0.0376 (6)	0.0253 (5)	−0.0235 (6)	−0.0240 (6)	0.0071 (4)
O2	0.0991 (13)	0.0576 (9)	0.0330 (6)	−0.0343 (9)	−0.0380 (8)	0.0094 (6)
O3	0.0959 (13)	0.0392 (7)	0.0502 (8)	−0.0366 (8)	−0.0420 (8)	0.0136 (6)

### Geometric parameters (Å, º)

**Table:**

C1—O2	1.215 (2)	C8—N1	1.417 (2)
C1—O1	1.367 (2)	C9—C10	1.373 (2)
C1—C2	1.434 (3)	C9—H9	0.9300
C2—C3	1.345 (2)	C10—H10	0.9300
C2—H2	0.9300	C11—O3	1.209 (2)
C3—C5	1.454 (2)	C11—N1	1.337 (2)
C3—C4	1.499 (2)	C11—C12	1.542 (2)
C4—H4A	0.9600	C12—F1''	1.23 (2)
C4—H4B	0.9600	C12—F1'	1.259 (19)
C4—H4C	0.9600	C12—F3	1.287 (8)
C5—C6	1.393 (2)	C12—F2	1.297 (8)
C5—C10	1.403 (2)	C12—F3'	1.327 (15)
C6—O1	1.3835 (17)	C12—F2''	1.327 (14)
C6—C7	1.384 (2)	C12—F1	1.343 (7)
C7—C8	1.388 (2)	C12—F2'	1.400 (18)
C7—H7	0.9300	C12—F3''	1.42 (2)
C8—C9	1.398 (2)	N1—H1	0.8600
			
O2—C1—O1	116.61 (16)	F1'—C12—F3'	111.2 (12)
O2—C1—C2	125.67 (16)	F3—C12—F3'	30.8 (9)
O1—C1—C2	117.72 (13)	F2—C12—F3'	84.4 (9)
C3—C2—C1	123.21 (15)	F1''—C12—F2''	114.2 (12)
C3—C2—H2	118.4	F1'—C12—F2''	134.9 (11)
C1—C2—H2	118.4	F3—C12—F2''	72.7 (13)
C2—C3—C5	118.12 (15)	F2—C12—F2''	42.9 (14)
C2—C3—C4	121.53 (15)	F3'—C12—F2''	42.7 (10)
C5—C3—C4	120.35 (14)	F1''—C12—F1	27.2 (16)
C3—C4—H4A	109.5	F1'—C12—F1	22.9 (14)
C3—C4—H4B	109.5	F3—C12—F1	105.9 (5)
H4A—C4—H4B	109.5	F2—C12—F1	106.3 (5)
C3—C4—H4C	109.5	F3'—C12—F1	129.8 (11)
H4A—C4—H4C	109.5	F2''—C12—F1	135.9 (10)
H4B—C4—H4C	109.5	F1''—C12—F2'	59.8 (14)
C6—C5—C10	116.77 (14)	F1'—C12—F2'	105.0 (11)
C6—C5—C3	118.39 (13)	F3—C12—F2'	123.9 (9)
C10—C5—C3	124.81 (14)	F2—C12—F2'	20.2 (10)
O1—C6—C7	115.01 (13)	F3'—C12—F2'	101.2 (11)
O1—C6—C5	121.47 (13)	F2''—C12—F2'	62.0 (14)
C7—C6—C5	123.52 (13)	F1—C12—F2'	87.0 (9)
C6—C7—C8	117.96 (14)	F1''—C12—F3''	104.1 (14)
C6—C7—H7	121.0	F1'—C12—F3''	59.8 (11)
C8—C7—H7	121.0	F3—C12—F3''	28.5 (10)
C7—C8—C9	120.26 (14)	F2—C12—F3''	135.5 (9)
C7—C8—N1	122.56 (14)	F3'—C12—F3''	59.3 (10)
C9—C8—N1	117.18 (12)	F2''—C12—F3''	100.8 (12)
C10—C9—C8	120.32 (13)	F1—C12—F3''	82.7 (15)
C10—C9—H9	119.8	F2'—C12—F3''	139.8 (12)
C8—C9—H9	119.8	F1''—C12—C11	120.0 (10)
C9—C10—C5	121.17 (14)	F1'—C12—C11	115.8 (9)
C9—C10—H10	119.4	F3—C12—C11	111.5 (4)
C5—C10—H10	119.4	F2—C12—C11	109.2 (4)
O3—C11—N1	127.88 (15)	F3'—C12—C11	109.6 (8)
O3—C11—C12	117.70 (15)	F2''—C12—C11	108.7 (5)
N1—C11—C12	114.40 (14)	F1—C12—C11	112.2 (4)
F1''—C12—F1'	47.4 (13)	F2'—C12—C11	113.0 (8)
F1''—C12—F3	119.8 (13)	F3''—C12—C11	106.8 (10)
F1'—C12—F3	84.0 (13)	C11—N1—C8	126.56 (12)
F1''—C12—F2	79.3 (15)	C11—N1—H1	116.7
F1'—C12—F2	122.1 (9)	C8—N1—H1	116.7
F3—C12—F2	111.5 (6)	C1—O1—C6	121.07 (13)
F1''—C12—F3'	130.4 (13)		

### Hydrogen-bond geometry (Å, º)

**Table:**

*D*—H···*A*	*D*—H	H···*A*	*D*···*A*	*D*—H···*A*
N1—H1···O2^i^	0.86	2.05	2.8960 (19)	170

Symmetry code: (i) *x*, *y*, *z*−1.
